# Suicide from a Holistic Point of View

**DOI:** 10.1100/tsw.2005.93

**Published:** 2005-09-13

**Authors:** Søren Ventegodt, Joav Merrick

**Affiliations:** ^1^ Nordic School of Holistic Medicine, Teglgårdstræde 4-8, DK-1452 Copenhagen K, Denmark; ^2^ Quality of Life Research Center, Teglgårdstræde 4-8, DK-1452 Copenhagen K, Denmark; ^3^ Quality of Life Research Clinic, Teglgårdstræde 4-8, DK-1452 Copenhagen K, Denmark; ^4^ National Institute of Child Health and Human Development, Faculty of Health Sciences, Ben Gurion University of the Negev, Beer-Sheva, Israel; ^5^ Center for Multidisciplinary Research in Aging, Faculty of Health Sciences, Ben Gurion University of the Negev, Beer-Sheva, Israel; ^6^ Division of Pediatrics, Faculty of Health Sciences, Ben Gurion University of the Negev, Beer-Sheva, Israel; ^7^ Office of the Medical Director, Division for Mental Retardation, Ministry of Social Affairs, Jerusalem, Israel

**Keywords:** Human development, suicide, public health, holistic medicine, Denmark, Israel

## Abstract

Suicide has been honoured and respected in the eastern culture, especially in Japan with the famous tradition of Hara-kiri, or seppuku, while in most western societies suicide has been seen negatively and many contemporary physicians tend to consider suicide the most self-destructive and evil thing a human being can do and something that should be avoided at all cost. Religions also have different viewpoints on suicide, but from a philosophical point of view we believe that considering the choice of life and dead to be extremely relevant for a good living. The choice of life and dead is real, since responsibility for life is necessary in order to live life and even the best physician cannot keep a patient alive, who deep inside wants to die. In this chapter, we present parts of a story of a young girl who had experienced child sexual abuse. In holistic existential therapy, it is our experience, when the patient is well supported in the confrontation of the fundamental questions related to assuming responsibility for the coherence, that this confrontation will almost always lead to a big YES to life. Without confronting the fundamental question of “to be or not to be”, life can never be chosen 100% and thus never be lived fully.

## INTRODUCTION

Suicide (from latin sui caedere, self killing) is the act of ending your life, which has been considered a sin and crime in many religions and societies. Some cultures, however, have viewed it as an honorable way to exit certain shameful or hopeless situations. Parasuicide is the term for "attempted suicide".

Suicide has been honoured and respected in the Eastern cultures with the famous tradition of Hara-kiri (called seppuku) as a well-known example. Hara-kiri is a ritual and honorable suicide with Japanese origins. Traditionally, it is done in a spiritually clean temple by cutting open your abdomen with a wakizashi (traditional Japanese sword with a shoto blade between 30 and 60 cm, with an average of 50 cm), thereby releasing the soul. The traditional form is one deep cut down and one across, while a slightly less honorable version (and much less painful) is that at the same time, a friend severs the head for an instant death. Hara-kiri was traditionally used as the ultimate protest, when your own morals stood in the way of executing an order from the master. It was also permissible as a form of repentance when one had committed an unforgivable sin either by accident or on purpose.

## HISTORY AND RELIGION

Many famous people through history, such as Cleopatra VII of Egypt, Hannibal, Nero, Adolf Hitler, Ernest Hemingway and Vincent van Gogh have committed suicide.

In Buddhism, the past influences our present and what an individual does in the present influences his, or her future, in this life or the next. This is cause and effect, as taught by Gautama Buddha. Known as karma, intentional action by mind, body or speech has a reaction and its repercussion is the reason behind the conditions and differences we come across in the world. Suffering primarily originates from past negative deeds, or just from being in samsara (the cycle of birth and death). Another reason for the prevalent suffering we experience is due to impermanence. Since everything is in a constant state of flux, we experience dissatisfaction with the fleeting events of life. To break out of samsara, one simply must realize his true nature through Enlightenment in the present moment; this is Nirvana. For Buddhists, since the first precept is to refrain from the destruction of life (including oneself), suicide is clearly considered a negative form of action. But despite this view, an ancient Asian ideology similar to Hara-kiri persists to influence Buddhists who, when under oppression, commit the act of "honorable" suicide. In Buddhism, suicide can be seen as an encouraged activity if it saves others (i.e., self-sacrifice in order to save others). Thus, it does not look at suicide from only a negative viewpoint.

Christianity is traditionally opposed to suicide and assisted suicide, and especially in Catholicism, suicide has been considered a grave and sometimes mortal sin. The chief Catholic argument is that your life is the property of God and to destroy your life is to assert dominion over what belongs to God. Many Christians believe in the sanctity of human life, a principle which, broadly speaking, says that all human life is sacred - a wonderful, even miraculous creation of the divine God - and every effort must be made to save and preserve it whenever possible.

In Islam, God is creator, he is the giver of life, and he alone has the right to end it. Suicide is forbidden in Islam and listed as a sin among the "enormities" in Reliance of the Traveller, a manual of Sharia in the tradition of Imam Shafi'i. Those who commit suicide should be *roasted in a fire* (do not kill yourselves, for Allah is compassionate towards you. Whoever does so, in transgression and wrongfully, We shall roast in a fire, and that is an easy matter for Allah. (an-Nisaa 4:29-30)) Forbidden Paradise (the Prophet said, "Whoever intentionally swears falsely by a religion other than Islam, then he is what he has said, (e.g. if he says, 'If such thing is not true then I am a Jew,' he is really a Jew). And whoever commits suicide with a piece of iron will be punished with the same piece of iron in the Hell Fire." Jundab, the Prophet, said, "A man was inflicted with wounds and he committed suicide, and so Allah said: My slave has caused death on himself hurriedly, so I forbid Paradise for him," (Sahih Bukhari 2.445) “and he will be punished in hell by whatever used for suicide.” The Prophet said, "He who commits suicide by throttling shall keep on throttling himself in the Hell Fire (forever) and he who commits suicide by stabbing himself shall keep on stabbing himself in the Hell-Fire." (Sahih Bukhari 2.446, 2.445).

Judaism views suicide as one of the most serious of sins. Suicide has always been forbidden by Jewish law, except for three specific cases: if one is being forced by someone to commit murder, forced to commit an act of idolatry, or forced to commit adultery or incest. However, outside of those cases, suicide is forbidden, and this includes taking part in assisted suicide. One may not ask someone to assist in killing themselves for two separate reasons: (a) killing oneself is forbidden, and (b) one is then making someone else an accomplice to a sin.

## WESTERN SOCIETY

In most western societies suicide has mostly been seen negatively. Many contemporary physicians tend to consider suicide the most self-destructive and evil thing a human being can do and something that should be avoided at all cost. Even the patient's contemplation of suicide is often considered harmful and treated as a disease in itself.

From a philosophical point of view, considering the choice of life and death is extremely relevant to good living and a very sound consideration. The choice of life and death is real, since responsibility for life is necessary in order to live life. Even the best physician cannot keep a patient alive who deep inside wants to die.

The reason for the western suicide is normally bad thriving as the tendency to have suicidal thoughts are closely connected to poor quality of life, or a feeling of having no value, or even feeling harmful to the surrounding world. One in 20 of the Danish population is at a given point in time considering suicide [1].

## HOLISTIC MEDICINE

In the process of holistic medical treatment, the physician, or therapist, must some times use a deep existential rehabilitation process[2], which can include a crisis where the patient will consider committing suicide[3] This danger must be considered as real. On the other hand, the deep contemplation of suicide and the following unconditional choice of life seem to be extremely beneficial for the patient deeply involved with personal development. This turns our view of suicide upside down and forces us to analyse the phenomenon of the urge for suicide (or the urge for death, as described by Freud[4]) in the existential perspective of healing the "soul", or core of existence. Freud stated that the subconscious of man contains two fundamental forces: a life force (or sexual force) and a death force. The life mission theory states that to annulate a painful life purpose one must intend destruction of self and others[5]. The logic is that we deepest down in existence want to do well. In situations where this is too difficult the emotional pain motivates us to deny this positive purpose of life. The only way to do so is to repress it by intending the opposite of the purpose, which is a negative and destructive intent. This dynamic seems to be highly active in early childhood. It is important to note that some existential thinkers like Arthur Schopenhauer (1788-1860) looked at life from a pessimistic angle, while holistic medicine is based on a positive perspective of life.

To understand the process of healing, the concept of "peeling the onion" is relevant[6]. In therapy, the patient digs deeper and deeper down the historical layers of traumas, and deeper and deeper into the heart of being. Three layers of existence can be identified, as they reveal themselves during holistic existential therapy (Figure 1):

1) the layer of global quality of life (QOL), mental/physical health and ability
2) the layer of love, power/consciousness and sexuality
3) the layer of existential coherence, where life inside the human being coheres with the outside world

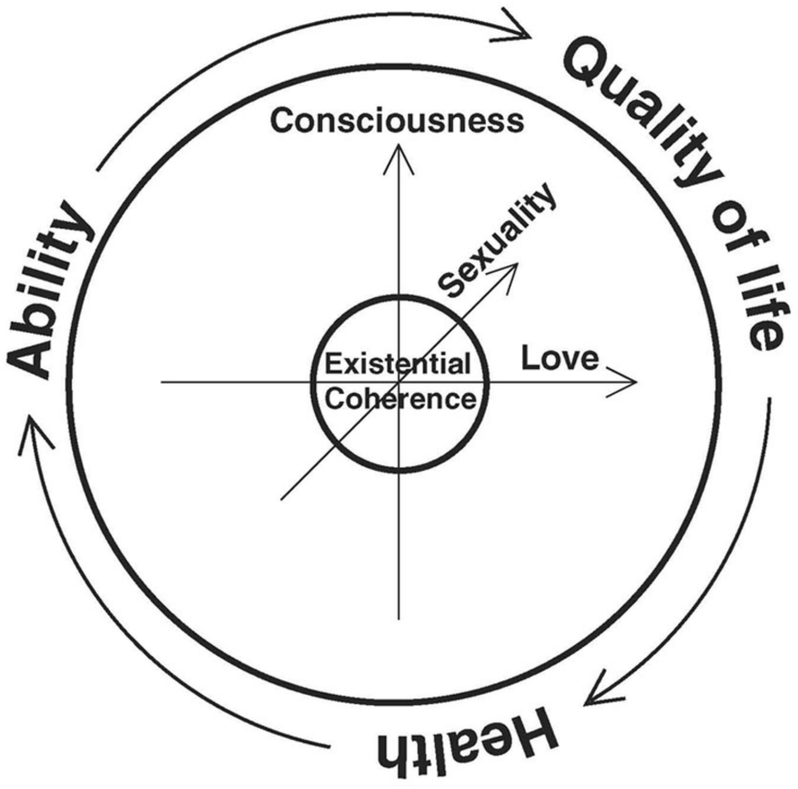

**FIGURE 1.** The human existence consists of three layers: 1) the layer of global quality of life (QOL), mental/physical health and ability, 2) the layer of love, power/consciousness and sexuality, and 3) the layer of existential coherence, where life inside the human being coheres with the outside world. When the patient, or spiritually seeking person, goes still deeper towards the innermost core of existence (s)he will one day confront the most fundamental question in life: To be, or not to be. This is the birth of the suicidal crises, which can only be definitely terminated by the patient deciding unconditionally to live. This decision must be taken autonomously, that is without any kind of pressure of external motivation.

Normally, what brings the patient to the clinic is a problem with one of the major issues of the first layer: quality of life – the patient is unhappy, stressed or in a crisis; health – the patient is sick, in pain or mentally disturbed or depressed, or ability – the patient is poor functioning and of little value to self or others. Searching for the causes of the problems in this layer normally will bring the patient to the second, inner layer of existence, where often the disturbances in the dimensions of love, understanding and gender/character can be seen as the disease causing the problems of health, QOL and ability. The problems of love, consciousness and sexuality are often emotionally overwhelmingly painful, and only with great support and intense "holding" will the roots of the existential imbalances be healed in the holistic medical clinic.

As the therapy goes still deeper, one day a third layer of existence is revealed. This layer seems to contain the core of existential responsibility. We call this innermost layer "coherence", or existential coherence[7], or Antonovsky coherence after the researcher naming it first (Aaron Antonovsky, 1923-1994)[8,9]. The problem of this layer is that it cannot really be processed in therapy as the question is: Do I want to be connected to the world? Do I want to be a part of this world? Do I want to live, or do I want to die? So we are back at the famous core question of existence, so beautifully worded by William Sheakspeare (1564-1616)[10]: "To be, or not to be".

The value of our autonomy is our free will; the prize of it is our loneliness. Only I can say if I want to live. So in the course of personal development, people who seek all the way to the basis of existence will face this question: When it comes down to it, do you want to live or do you want to die? The urge to die is very logical at this level: to be connected is too painful, to reach for the meaning of life is too much for me, to be is connected with feelings and emotions too unbearable to be acceptable. The urge to live is just as logical: I am here as a gifted person, and the meaning of my life is to share my gift with other people and the world in order to create value as I was meant to.

The internal struggle on life and death that follows from these contradictory and opposing forces in the root of every individual human soul is, from this perspective, what causes the suicidal crises of man. Interestingly, therefore, the suicidal crisis becomes extremely important in the process of healing with the understanding and wise handling of the suicidal patient, guiding the patient towards confronting the fundamental existential question, “To be, or not to be”. This question is what brings growth, learning and personal progress to the troubled patient.

## A CASE HISTORY

Anna was a student, aged 22 years, who had completely repressed over 100 episodes of sexual abuse, incest and rape throughout her early childhood. She now seems to have recovered completely, including having regained her full emotional range through holistic existential therapy, individually and in a group. The therapy took 18 month and more than one hundred hours of intensive therapy. In the beginning of the therapy, the issues were her physical and mental health; in the middle of the therapy the central issue was about her purpose of life and her love life, and in the end of the therapy, the issue was gender and sexuality. The strategy was building up her strength for several months, mobilizing all her hidden resources and motivation for living, before the painful old traumas were confronted and integrated.

The following is from her case diary. Just before she ended her therapy, the suicidal crisis came and choosing life, at the end of this crisis, was what gave her the final breakthrough to life and survival[3].

> **Saturday**
> After a nice bath, I dressed and got ready to go out enjoying the nature. To begin with I must say that the sun had been shining on me the whole day; so beautiful and fine it was that I couldn’t help saying hello, while warming me on the outside and on the inside. This grew into many greetings. I was down near the Marienlyst Castle and was sitting at the end of the avenue, of course with the sun on my chin. The place was really beautiful and minimalistic. I proceeded a bit towards Helsingoer, but it wasn’t the right way; too many cars, houses and first and foremost, too much noise. Therefore I went back to Hellebaek and followed the beach with my recently purchased goods in the bag: chips and white bread, uhmmm. How great an outing it had been! I could sense how I got calmed and at the same time boosted from listening to the roar of the waves. I sat down on a big stone, on the cushion I had brought along, enjoyed the sound of the water and shuddered slightly at the warm sunbeams. While sitting there I thought of it once again: that I am an unwritten leaf and that right now I am exceptionally lucky, because I have got the chance of shaping my life and myself like I want it most. Now I can cultivate the capacities, skills and qualities I greatly prefer to possess, and this without the usual, rotten wreckage in tow, which could prevent me from doing it. There is a huge opportunity right now! I was also thinking this means that now I will actually be capable of getting/achieving ANYTHING I want. I just have to set about getting/achieving it. I walked to Hellebaek, about 6 km, along the beach and walked back via the woods. I thought of the song “The woods around the country are turning yellow now”, and changed the title into “The woods around the country are glowing now”, because I found that was what they actually did. The woods were unbelievably beautiful right now, in fact, got my eyes filled with tears. It’s indeed a huge gift for me to be up here in the beautiful nature. It was, no doubt, the completely right impulse to follow! As I returned here I began to paint/colour a bit in the colouring book. However, it didn’t really mean anything to me though, so I didn’t finish the drawing. It was so boring … Then I laid down to rest. I dreamed a little, but as I didn’t manage to maintain the dream, I forgot it again. Now I have been taking a bath thus, and I want to read a little before going to sleep again. Tomorrow I shall check out at 9:30 a.m. I am curious to know whether I'll wake up, having got no watch. What I have been experiencing has been right: To stay in the pleasure: going for a walk today, giving myself fully into the pleasure; not holding back at all and being able to stay in it. To give myself for: the anger. The same principle as for the pleasure: not holding back, being one hundred percent in it. - This is to be alive!

**Saturday night**

I twist about miserably in my bed, sweat as if I had got a very high temperature. The anger is huge and while lying here I am full of it. I beat the mattress, swear, snub them, and then begin projecting anger onto Søren (the therapist/physician). I get angry with him at the way he treated me this week; the rough way mixed with an apparent indifference as to the way he had been reflecting me. Then the suicide thoughts appeared:

- pistol: too much mess and too traumatic for those who find me
- cut my throat: same thing as with the pistol
- cut the wrists: then I shall suffer too long
- liquidation: I could pay someone to do it; this one is the best I immediately find until I am thinking of:
- overdose: which would be much better. A second later I think that I would probably not hit the correct dose, but would brain-damage myself and end as a vegetable, dribbling and not even being able to communicate to people that they must kill me. Then Søren would call on me, hold me close, and this would be the ultimate hell; me not being able to communicate, only dribble.

Now I cry and am totally miserable. I still sweat fever. I think then: Stop – just be quiet. One day at the time. I say aloud: "I bring life and joy. I bring life. I bring life" quite a lot of times and this calms me; this slowly makes me to relax. [Anna is here assuming responsibility for her own existence at the most deepest level; she is facing the need of choosing to live or to die, accepting life on its own conditions or not accepting it. This is really the deepest level of existential choice for any human being: do you want to live or do you want to die? And it is a strictly personal question; nobody can really help you out here, you need to solve this for yourself, as Anna instinctively did.]

**Monday**

At long last I had a decent conversation with Søren. I had hurt his feelings, made him sad. He said I did it to create a distance between us. I told him that I was fond of him. He thanked and finished the conversation saying he was fond of me too. Subjects: detachment, independence. I slept very bad tonight, Søren and our understanding dialogue about "what did happen" the last few days being constantly in my thoughts. I even wrote a poem while shifting about restlessly.

**Tuesday**

I was at my gestalt therapist today. Further, I am thinking that if I play my cards well I can end as something big. With my story, my intuitive intelligence and my courage I think I can become an entirely tough therapist. Watch me! Later I talked with Søren; he was making fun and said I would soon be able to take my gestalt therapist in therapy. It was funny said, and I must admit that later I will be forgetting her face while telling her how I had been experiencing my therapy. Not only was she gaping, she also realized that she was facing a very intelligent girl who had just discovered how intelligent she was. An educational experience, indeed! Let me finish here by mentioning that my personal development will no doubt carry on. I have been releasing so amazingly much insecurity. Never before have I been feeling so confident that everything will turn out all right. I find I keep on getting ever more gorgeous, and I am sure I shall get the best boyfriend in the whole world. I am in the process of being quite happy; I am not miserable any more. I am convinced I shall become entirely happy.

## DISCUSSION

Fear of letting the patient confront the deep existential pain of loneliness and low coherence often makes the physician use force to save the patient's life. Often the physician uses strong anti-psychotic drugs and conversational therapy of a cognitive type to turn the patient's attention outwards against the outer world and away from the existential problems, thus avoiding the patient's confrontation of the emotional pain and the temptation of the suicidal perspective.

Our study implies that the current training of physicians is not quite adequate. Physicians should be better trained to understand the basic existential conditions of man: anxiety rising from being mortal, loneliness from being autonomous and suffering from being a creature of love, wanting so desperately to contribute to the world at large in spite of the vast difficulties in this for every human being. To confront the deep existential pain of loneliness and coherence which exist deep down in the soul of every human being, it is necessary for the physician to engage himself in existential therapy and personal development for at least a couple of years.

From a holistic perspective, this approach only deals with the symptoms of the real disease, which is lack of existential coherence, and can thus not be recommended as the problem stays with the patient and can surface anytime again leaving the patient in tremendous danger of actually committing the suicide the physician so eagerly tries to prevent. There is always a risk that the patient, during the process of confronting the original painful causes of the existential disconnection, can waste his or her own life if not sufficiently supported. In holistic existential therapy, it is our experience that when the patient is well supported in the confrontation of the fundamental questions related to assuming responsibility for the coherence, that this confrontation will almost always lead to a big YES to life. Without confronting the fundamental question of "to be, or not to be", life can never be chosen 100% and thus never be lived fully.

The dynamics of suicide, wanting to die so much that self-destruction of the body becomes an issue, is the most painful experience a human being can go through. On the other hand, it seems that solving the existential problems at the deepest level is the most beneficial achievement of all and the most important thing a person can be supported in doing. The taboo of death in most western cultures has turned an existentially sound quest for meaning and search for healing of the soul and reconnection to the world into something shameful and bad, something that is often condemned. With a more profound, honest and brave understanding of life and personal growth, the reflection on suicide can be changed from being something we in our society and medical facilities try to avoid at all cost, to something natural and beautiful that we as holistic physicians and health professionals must support and guide our patients through.

The intense suffering connected to the dynamics of suicide is something we cannot spare the patient, but we can give him the benefit of his hard work, which only becomes obvious when the choice of life and death is real and life is finally chosen unconditionally. With a lot of "holding" and caring support even the most painful feelings of being completely worthless and unwanted on this planet are easier to deal with, contain and finally integrate for the patient.

## References

1. Ventegodt, S. (1995) Quality of life in Denmark. Results from a population survey. Copenhagen: Forskningscentrets Forlag. [Danish]
2. Ventegodt, S., Gringols. M,, and Merrick, J. (2005) Clinical holistic medicine: Holistic rehabilitation. *TheScientificWorldJOURNAL*. **5**, 280-287.
3. Ventegodt, S., Clausen, B., and Merrick, J. Clinical holistic medicine: The case story of Anna. II Patient diary with the holistic process of healing seen from within the patient. Submitted to *TheScientificWorldJOURNAL*.
4. Freud, S., and Bril,l A.A., ed. (1936) The basic writings of Sigmund Freud. New York: Modern Library.
5. Ventegodt, S. (2003). The life mission theory: A theory for a consciousness-based medicine. Int. J. Adolesc. Med. Health. **15(1)**, 89-91.
6. Perls, F., Hefferline, R., and Goodman, P. (1951) Gestalt therapy. New York: Julian Press.
7. Ventegodt, S., Flensborg-Madsen, T., Andersen, N.J., and Merrick, J. (2005). Life Mission Theory VII: Theory of existential (Antonovsky) coherence: a theory of quality of life, health and ability for use in holistic medicine. *TheScientificWorldJOURNAL*. **5**, 377-389.
8. Antonovsky, A. (1985) Health, stress and coping. London: Jossey-Bass.
9. Antonovsky, A. (1987) Unravelling the mystery of health. How people manage stress and stay well. San Franscisco: Jossey-Bass.
10. Alexander, P. (1991) William Sheakspeare: The complete works. London: Collins.

## BIOSKETCHES

**Søren Ventegodt, MD**, is the director of the Quality of Life Research Center in Copenhagen, Denmark. He is also responsible for a Research Clinic for Holistic Medicine in Copenhagen and noted as a popular speaker throughout Scandinavia. He has published numerous scientific or popular articles and a number of books on holistic medicine, quality of life and quality of working life. His most important scientific contributions are the comprehensive SEQOL questionnaire, the very short QoL5 questionnaire, the integrated QOL theory, the holistic process theory, the life mission theory, and the ongoing Danish Quality of Life Research Survey, 1991-1994 in cooperation with the University Hospital of Copenhagen and the late professor of pediatrics, Bengt Zachau-Christiansen, MD, PhD. E-mail: ventegodt@livskvalitet.org. Website: www.livskvalitet.org

**Joav Merrick, MD, DMSc**, is professor of child health and human development affiliated with the Center for Multidisciplinary Research in Aging, Zusman Child Development Center, Division of Pediatrics and Community Health at the Ben Gurion University, Beer-Sheva, Israel, the medical director of the Division for Mental Retardation, Ministry of Social Affairs, Jerusalem, the founder and director of the National Institute of Child Health and Human Development. Has written numerous publications in the field of child health and human development, rehabilitation, intellectual disability, disability, health, welfare, abuse, advocacy, quality of life and prevention. Received the Peter Sabroe Child Award for outstanding work on behalf of Danish Children in 1985 and the International LEGO-Prize (“The Children’s Nobel Prize”) for an extraordinary contribution towards improvement in child welfare and well-being in 1987. E-Mail: jmerrick@internet-zahav.net. Website: www.nichd-israel.com
